# The use of haemopoietic heterochimeras for the detection of leukaemogenic virus.

**DOI:** 10.1038/bjc.1968.20

**Published:** 1968-03

**Authors:** G. Mathé, R. Motta, S. Sorieul

## Abstract

**Images:**


### THE USE OF HAEMOPOIETIC HETEROCHIMERAS FOR THE DETECTION OF LEUKAEMOGENIC VIRUS

G. MATHÉ, R. MOTTA AND S. SORIEUL

*From the Institut de Cancérologie et d'Immunogénétique, Hôpital Paul-Brousse, 14 Avenue Paul-Vaillant-Couturier, 94-Villejuif, France*

Received for publication November 8, 1967

SEVERAL carcinogenic viruses, known to be responsible for spontaneous tumours in a given species, are able to induce a malignant transformation either *in vitro* or *in vivo* in the cells of an animal of another species. Yet their discovery has always been made by their inoculation into animals of the same species in which they had induced a so-called spontaneous tumour. This applies particularly to the Gross virus, the only virus whose role in spontaneous leukaemogenesis in mice is certain (Gross, 1961).

It would seem that the greatest chance of revealing a leukaemogenic virus, in a material which only contains a small amount, would be by injecting animals of the same species as those in which it had appeared, rather than by injecting into other species. It is reasonable to think that there would be a greater chance of revealing a possible leukaemogenic virus in man if the biological material being studied were to be injected into human beings rather than into animals. These experiments in a direct form are not ethically acceptable, but an indirect approach could be used by injecting human material into animals with functioning human haemopoietic tissue—animal human chimeras.

Furthermore, it must be remembered that the detection of viruses responsible for spontaneous tumours is often facilitated by using immuno-incompetent hosts, which are usually new-born animals. In haematopoietic chimeras there also exists an immune incompetence (Mathé, Amiel and Daguet, 1961), which is another reason for using this system. Before attempting to form haemopoietic chimeras between man and monkeys, we studied the possibility of detecting a known leukaemogenic virus in mice by inoculation of rat-mice haemopoietic chimeras. This was preceded by experiments in which the leukaemogenic viruses of the Friend (1957) or Rauscher (1962) type were demonstrated by their injection into animals with an allogeneic chimera of the haemopoietic system (Mathé and Amiel, 1966).

### MATERIALS AND METHODS

Four hundred and twenty rats, male and female, of the Wistar CF strain, aged 8 days, were irradiated with a dose of 800 rads total body irradiation (260 kv 0·5 mm./Cu, CDA 1·75 mm./Cu, distance 70 cm.). On the following day they were injected intravenously with $1 \cdot 5 \times 10^8$ bone marrow cells from (DBA/2 × C57Bl/6) $F_1$ hybrid mice (males or females) and 6 days after this transfusion they were injected intraperitoneally with 0·2 ml. of a solution of Friend virus (the supernatant from a 35 per cent w/v solution of spleen, centrifuged at 2500 r.p.m. for 20 minutes).

When the rats died spontaneously or when they were killed because their death appeared to be imminent, the chromosomes of bone marrow and the spleen were examined and a full macroscopic and microscopic examination made of the animals. The cytotoxicity of rat antisera against mouse cells and of mouse anti--sera against rat cells was studied on the bone marrow and spleen. The percentage of cells killed by each of these anti-sera in the same cell suspension allowed the percentage of each cell type in the mixture to be calculated. The technique used was that described by Bennett, Old and Boyse (1964). In each test the cytotoxic action of each anti-serum was checked on pure populations of lymphoid cells from rats and mice of the same strains as used in the chimeras. The anti-sera were taken from animals of the same strains as those used for the hetero-specific graft experiments.

When splenomegaly was observed $10^6$ spleen cells were inoculated into CF Wistar rats and (DBA/2 × BALB/c) $F_1$ hybrid mice of the opposite sex to the animals that had served as the donor of bone marrow to the rat on which the genetic identity of the spleen cells had been studied.

### RESULTS

Three hundred and twenty one rats died of aplasia, 57 died of the runt syndrome (Fig. 1) between the 20th and 50th day and in 42 animals leukaemia was observed between the 14th and 81st day (Fig. 2).

The establishment of a haemopoietic cell heterograft was proved easily as 100 per cent of the bone marrow cells in mitosis had mouse chromosome (Fig. 3), and by the study of the antigenicity of the marrow cells using the cytotoxic anti-sera. The runt syndrome provided further evidence of the active function of the graft.

Several pieces of evidence pointed to the fact that the leukaemic cells were of murine origin. The leukaemia was a hepatosplenomegalic leukaemia (Fig. 2) identical to that induced by the Friend virus in mice. It was an erythroblastic and monoblastic leukaemia and not a lymphocytic leukaemia. In the leukaemic animals, most of the cells in the spleen were involved in the leukaemic process and these were shown to be of murine type by chromosomal studies (100 per cent of the chromosomes were shown to be of the mouse type) and by the cytotoxicity test (Table I). Further evidence of the murine origin of the leukaemic cells was provided by the grafting experiments in which leukaemic cells from the spleens of the rat-mice chimeras, which received marrow grafts from female (DBA/2 × BALB/c) $F_1$ hybrid mice, were injected into adult intact CF Wistar rats and adult intact male (DBA/2 × BALB/c) $F_1$ hybrid mice. None of the 67 rats but all of the 18 mice developed leukaemia, and chromosome analysis of

---

EXPLANATION OF PLATES.

FIG. 1.—Wistar CF rats of the same age. The two smaller animals are suffering from runt disease following irradiation and grafting of haemopoietic cells from (DBA/2 × BALB/c) $F_1$ hybrid mice.

FIG. 2.—Macroscopic appearance of the leukaemia induced by Friend virus in the irradiated Wistar CF rat grafted with murine (DBA/2 × BALB/c) $F_1$ haemopoietic cells. Note the gross hepatosplenomegaly.

FIG. 3.—Cell karyotype of male rat and of female (DBA/2 × BALB/c) $F_1$ mouse.

FIG. 4.—Cell karyotype of male and female (DBA/2 × BALB/c) $F_1$ mouse.

1

2

FIG. 3

Mathé, Motta and Sorieul.

cell karyotype of male

DBA/2×BALB/c mouse.

I

II

III

IV

Y

Y

cell karyotye of female

DBA/2×BALB/c mouse

I

II

III

IV

Y

FIG. 4

TABLE I.—*Percentage of Cells of Mouse and Rat Origin in the Spleen of the Leukaemic Animals Determined by the Cytotoxicity Test*

| Chimera number | Rat cells % | Mouse cells % |
|---|---|---|
| 1304 | 11 | 100 |
| 1305 | 0 | 100 |
| 1327 | 0 | 100 |
| 1333 | 5 | 99 |
| 1334 | 11 | 80 |
| 1335 | 11 | 100 |

the spleen cells from the leukaemic mice showed that the majority were of female karyotypes (Table II).

TABLE II.—*Number of Male and Female Mitoses in the Spleens of Recipient Male Isogeneic Mice Developing Leukaemia After Injection of Leukaemic Spleen Cells from Wistar CF Rat–Mouse Chimeras. All the Rat Chimeras Had Been Grafted with Female Mouse Spleen Cells.*

| Mice | Female mitosis | Male mitosis |
|---|---|---|
| 986a | 6 | 3 |
| 986b | 5 | 4 |
| 1110a | 3 | 3 |
| 1110b | 2 | 3 |
| 1181 | 9 | 3 |
| 1183 | 0 | 0 |
| 1173 | 9 | 4 |
| 1162 | 0 | 0 |
| 1266 | 4 | 3 |
| 1304 | 4 | 2 |
| 1305 | 5 | 2 |
| 1296a | 1 | 1 |
| 1296b | 4 | 1 |
| 1311 | 2 | 1 |
| 1327 | 4 | 0 |
| 1333 | 3 | 1 |

This experiment showed that the leukaemia in the mice was largely due to the graft and not induced by the virus carried by the leukaemic cells, as the recipients (all males) developed a leukaemia in which the majority of the cells did not have a Y chromosome which can easily be identified (Fig. 4). This demonstrates that it is possible for cells, induced to become leukaemic by the Friend virus, to establish themselves as a graft, as well as inducing leukaemia in the cells of the grafted host due to the virus present in the leukaemic cells, In our experiments the cells of the donor and the host could be distinguished by their sex chromosomes.

The system we have proposed would appear to be a very sensitive method to detect a leukaemogenic virus in a species, without having to use animals of that species and cause them to run the risk of contracting the leukaemia. We now consider that this system merits use in the search for leukaemogenic viruses in man, using a heterochimera of the human haemopoietic cells in a monkey.

### SUMMARY

A system is proposed which uses haemopoietic chimeras to detect a leukaemogenic virus. The present work has demonstrated that Friend virus can be demonstrated in a rat-mouse heterospecific chimera. It is suggested that chimeras of human haemopoietic cells in the monkey might be used to detect leukaemogenic viruses in man.

This work was done with the aid of INSERM, contract n°. CR.66–235.

### REFERENCES

BENNETT, B., OLD, L. J. AND BOYSE, E. A.—(1964) *Transplantation*, **2** 183.
FRIEND, C.—(1957) *J. exp. Biol.*, **105**, 307.
GROSS, L.—(1961) 'Oncogenic viruses', London (Pergamon Press) Vol. 1.
MATHÉ, G. AND AMIEL, J. L.—(1966) *C.r. hebd. Séanc. Acad. Sci., Paris*, **262**, 1323.
MATHÉ, G., AMIEL, J. L. AND DAGUET, G.—(1961) *Nouv. Revue fr. Hémat.*, **1**, 650.
RAUSCHER F. J. A.—(1962)*J. natn. Cancer Inst.*, **29**, 515.


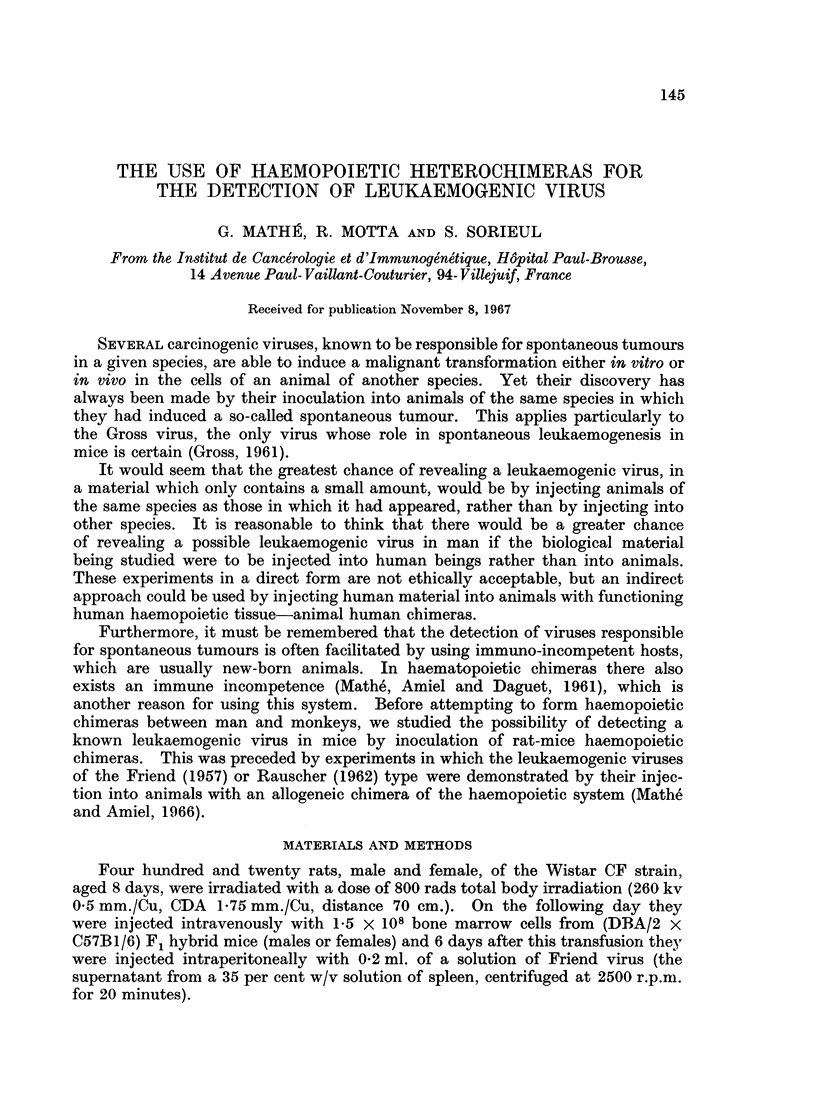

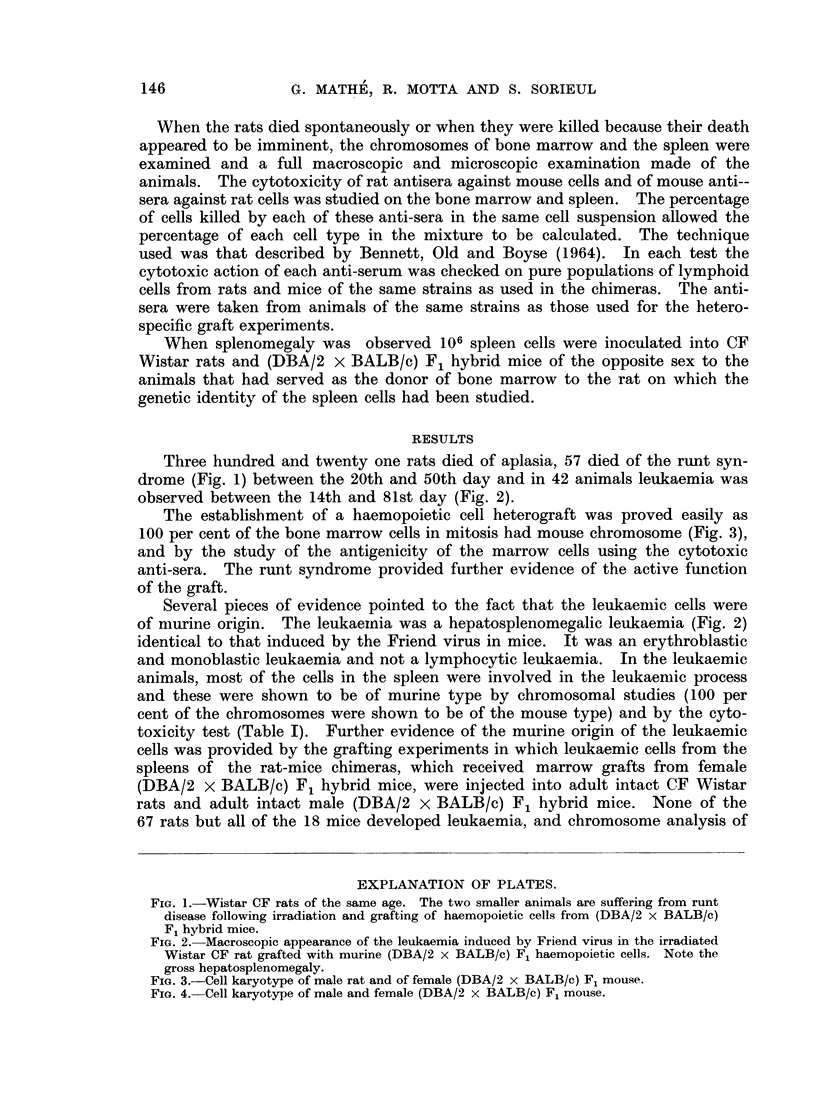

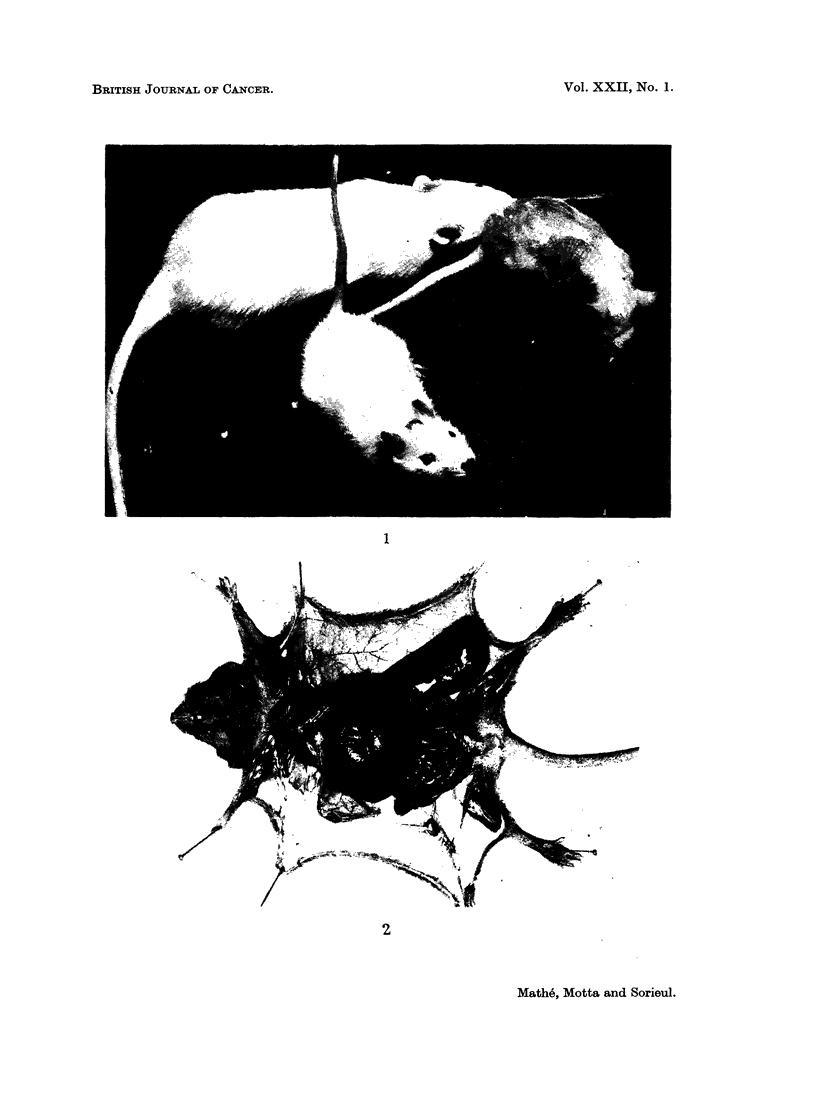

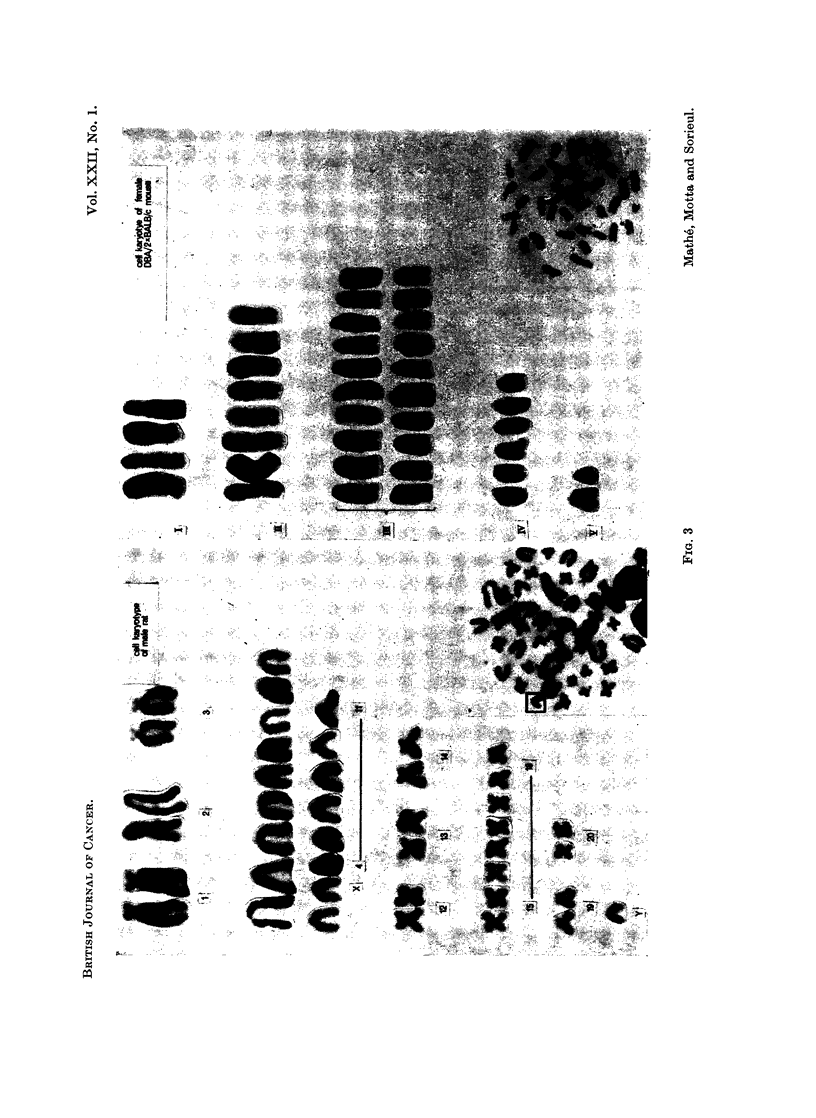

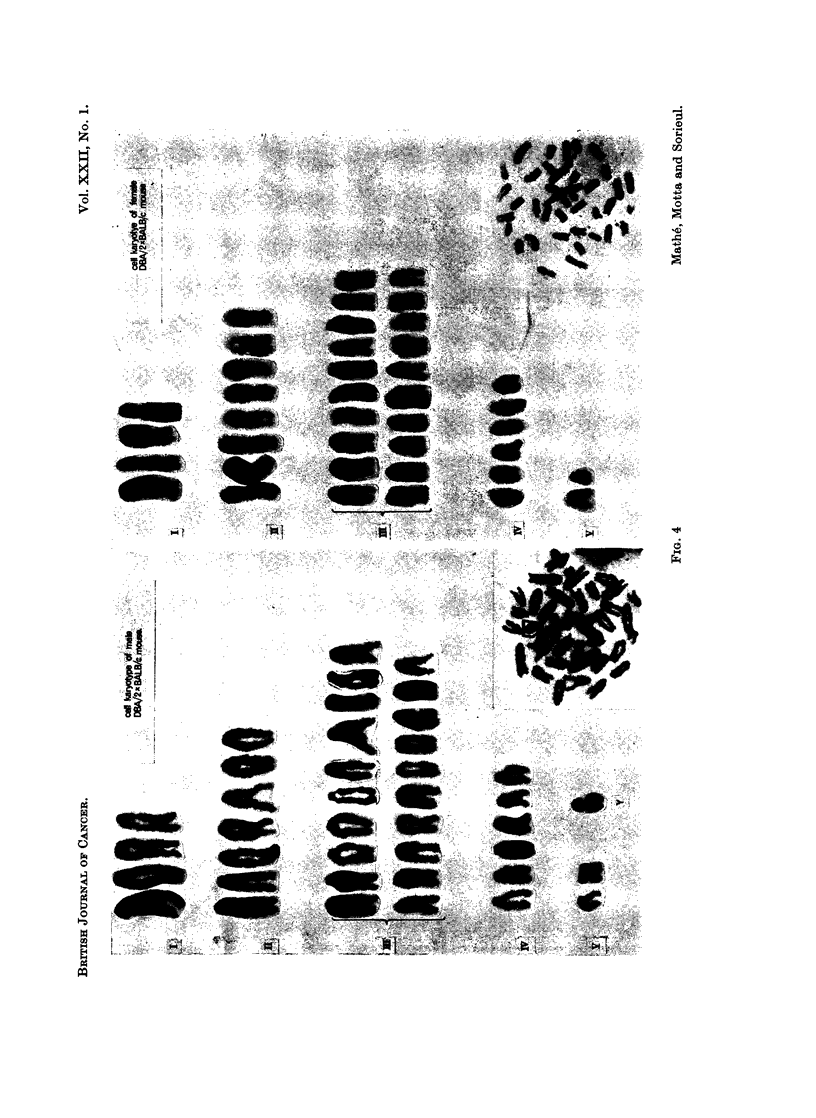

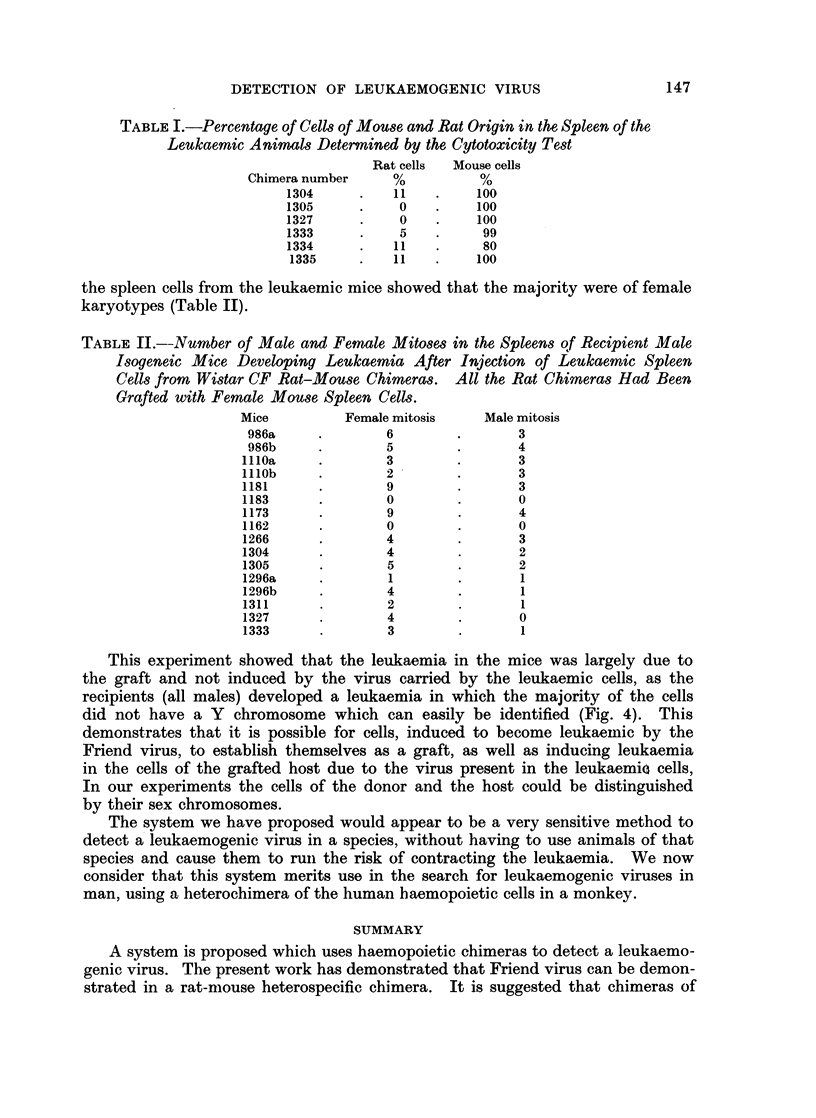

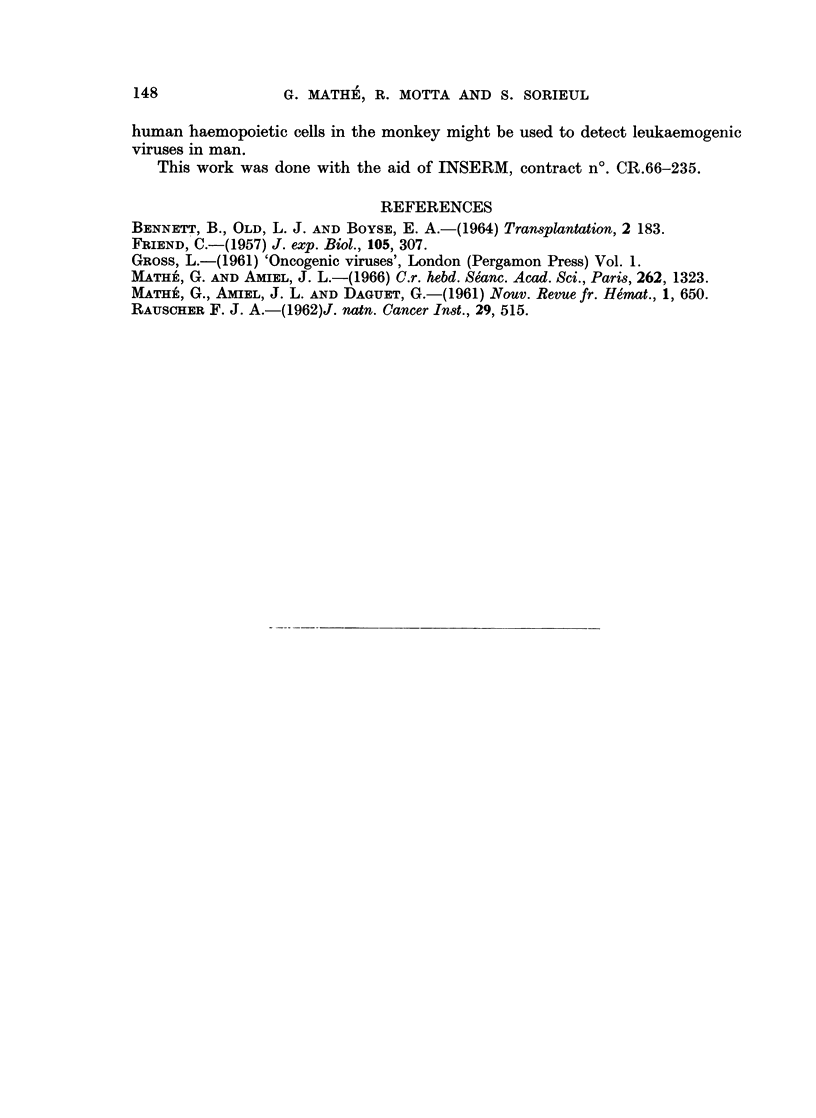


## References

[OCR_00501] BENNETT B., OLD L. J., BOYSE E. A. (1964). THE PHAGOCYTOSIS OF TUMOR CELLS IN VITRO.. Transplantation.

[OCR_00502] FRIEND C. (1957). Cell-free transmission in adult Swiss mice of a disease having the character of a leukemia.. J Exp Med.

[OCR_00506] Mathé G., Amiel J. L. (1966). Révélation de virus leucémigènes grâce au chimérisme hématopoïtique. Chimérisme allogénique chez la souris.. C R Acad Sci Hebd Seances Acad Sci D.

[OCR_00508] RAUSCHER F. J. (1962). A virus-induced disease of mice characterized by erythrocytopoiesis and lymphoid leukemia.. J Natl Cancer Inst.

